# ORY-1001 Suppresses Cell Growth and Induces Apoptosis in Lung Cancer Through Triggering HK2 Mediated Warburg Effect

**DOI:** 10.3389/fphar.2018.01411

**Published:** 2018-12-04

**Authors:** Zhaoliang Lu, Yanke Guo, Xiaoya Zhang, Jing Li, Leilei Li, Shuai Zhang, Changliang Shan

**Affiliations:** ^1^The First Affiliated Hospital, Biomedical Translational Research Institute, Jinan University, Guangzhou, China; ^2^State Key Laboratory of Medicinal Chemical Biology, College of Pharmacy and Tianjin Key Laboratory of Molecular Drug Research, Nankai University, Tianjin, China; ^3^School of Integrative Medicine, Tianjin University of Traditional Chinese Medicine, Tianjin, China; ^4^Department of Medical Biochemistry and Molecular Biology, School of Medicine, Jinan University, Guangzhou, China

**Keywords:** lysine (K)-specific demethylase 1A, Warburg effect, Hexokinases 2, cell proliferation, lung cancer

## Abstract

ORY-1001, an inhibitor of covalent lysine (K)-specific demethylase 1A (KDM1A), has been used as a therapy for the treatment of acute leukemia. However, the underlying mechanisms of anticancer are still not fully elucidated. Here, we report that KDM1A is highly expressed in lung cancers, where it appears to drive aggressive growth. Furthermore, lung cancer patients with higher KDM1A levels have worse survival outcomes than patients with lower KDM1A levels. Interestingly, ORY-1001significantly inhibited the cell proliferation, colony formation, cell cycle, and induced apoptosis, by regulating the Warburg effect through controlling Hexokinases 2 (HK2) expression. In summary, these results indicate that ORY-1001 could inhibit the growth of lung cancer cells via regulating the Warburg effect by controlling HK2.

## Introduction

Energy metabolism reprogramming is a hallmark of cancer. Cancer cells increase glucose uptake, which then leads to an increase in the aerobic glycolysis rate and enhances lactate and energy production, known as the Warburg effect (Cairns et al., 2011; Fan et al., 2014). Mounting evidence supports that deregulated energy metabolism reprogramming is closely related to drug resistance in cancer therapy (Liu et al., 2008; Wang et al., 2010; Zhou et al., 2010; Zhao et al., 2011, 2013; Zheng et al., 2017). The enzymes involved in energy metabolism reprogramming can be regulated at the gene expression and post-translation modification level, in response to extracellular and intracellular signaling, to cope with the adaptive metabolic remodeling, in response to severe environmental conditions. Blocking the epigenetic regulation of metabolic enzymes is not only an intellectual pursuit, but also provides a way to help diagnose and treat cancers.

Lysine (K)-specific demethylase 1A (KDM1A), also known as Lysine-specific demethylase-1 (LSD1), is the first reported histone demethylase, that can remove histone H3 Lys 4 and Lys 9′s mono- and di-methylation modification (Sehrawat et al., 2018). KDM1A has been characterized as a potential oncogene and a therapeutic target in various cancers (Hayami et al., 2011; Schildhaus et al., 2011; Yu et al., 2013). Several studies showed that various cancers with higher KDM1A expression correction, have high cell proliferation rates (Lv et al., 2012; Zhao et al., 2012; Jie et al., 2013; Yu et al., 2013). In acute myeloid leukemia (AML), KDM1A overexpression blocks differentiation and results in a poor prognosis (Fang et al., 2017; Fiskus et al., 2017). Sakamoto et al. (2015) recently found that KDM1A suppresses murine adipocytes mitochondrial respiration and maintains energy storage under obese conditions (Sakamoto et al., 2015). Targeting KDM1A by small molecule inhibitors, blocks cell proliferation in leukemia (Magliulo et al., 2018). It is therefore interesting to test whether targeting KDM1A, would block metabolic reprogramming in cancer cells driven by KDM1A. To better understand tumorigenesis, we must understand the metabolic reprogramming of cancerous cells. For this reason, the cancer metabolic reprogramming driven by KDM1A, has attracted much attention. Nevertheless, the precise contribution of KDM1A to cancer metabolism remains unclear.

Maes et al. have developed ORY-1001, a potent and selective inhibitor of KDM1A, for the inhibition of acute leukemia cell growth (Maes et al., 2018). But, the anti-cancer activity of ORY-1001 in lung cancer is unknown. In this study, we reported that KDM1A is highly expressed in lung cancer tissues and lung cancer cells and regulated cell proliferation. Furthermore, we found that ORY-1001 inhibited lung cancer cell proliferation, cell cycle and induced apoptosis by triggering the Warburg effect, by regulating HK2 expression. These results suggest the hypothesis that cancer cells are transmitted more on the glycolytic pathway than normal cells and targeting KDM1A may represent a promising approach for selectively causing cell proliferation in cancer cells.

## Materials and Methods

### Cell Lines

H1299, H157, H1944, H226, and H460 cells were cultured in a RPMI 1,640 medium with 10% FBS at 37°C and 5% CO_2_. The human lung adenocarcinoma epithelial cells A549, were gifted by Dr. Zhi Shi (Jinan University, Guangdong, China). The A549 cells were cultured in a Dulbecco Modified Eagle Medium (DMEM) with 10% FBS. Normal proliferating Human Bronchial Epithelial Cell Line (BEAS-2B) was gifted by Dr. Chenglai Xia (Guangzhou Medical University, Guangdong, China) and were cultured in a RPMI 1,640 medium with 10% FBS at 37°C and 5% CO_2_.

### Cell Proliferation Assay

For cell proliferation assay, 5 × 10^4^ cells were seeded into 6-well plates and cultured at 37°C and 5% CO_2_ for 12 h. The cells were then treated with ORY1001, with an increased concentration (80 and 160 μM) or vehicle alone for 1, 2, 3, and 4 days, and the cell number was counted.

### Colony Formation Assay

Eight hundred cells (H1299 and A549) were plated into 6-well plates and were cultured in a RPMI 1,640 or DMEM medium at 37°C and 5% CO_2_, in a humidified incubator. The cells were then replenished with a fresh RPMI 1,640 or DMEM medium containing ORY1001 (80 and 160 μM) after 10 days and incubated for another 5 days. The treated cells were washed with pre-warmed PBS three times, fixed with methanol for 20 min, and stained with crystal violet for 15 min. The residual crystal violet was then removed using double distilled H_2_O, and the plates were then air-dried. The colony numbers were counted using Image plus software. Each experiment was assayed in triplicate with three independent experiments.

### Apoptosis Assay

Cell apoptosis was determined by flow cytometry. Firstly, the cells were harvested and washed with PBS. Subsequently, an Annexin V fluorescein (FITC)/propidium iodide (PI) double staining solution was used on the cell sample, to detect apoptosis following the manufacturer's protocol. The samples were analyzed using the BD FACScalibur flow cytometer (BD Biosciences), and subsequent analyses were performed with FlowJo software. All assays were performed in triplicate.

### Cell Cycle Assay

H1299 and A549 cells were seeded into 6-well plates and treated with 80 and 160 μM ORY1001 for 48 h. The cells were then harvested, washed with PBS, centrifuged and fixed in cold 70% ethanol at 4°C for 12 h. The samples were washed with PBS after fixation to remove the ethanol. Subsequently, PBS containing 10 mg/mL propidium iodide (PI; Sigma-Aldrich) and RNase A (100 mg/mL, Solarbio) was added to the cell sample, at room temperature for 10 min under darkness. Finally, the samples were analyzed using the BD FACScalibur flow cytometer (BD Biosciences).

### Western Blotting

Cells were lysed with lysis buffer [1.5 M NaCl, 1 M HEPES[pH = 7.0], 1%NP40, 0.1 M Na4P2O7, 0.1 M NaF, 0.1 M Na3VO4, protease inhibitor] on ice for 30 min and then centrifuged at 12,000 rpm for 15 min at 4°C. Protein samples were loaded into 12% SDS-PAGE, then separated by running different Voltages, and transferred onto PVDF membranes (Millipore). The membranes were blocked with 5% non-fat milk for 2 h and then incubated overnight at 4°C, or at room temperature for 2 h with the primary antibody and for 1 h at room temperature with the secondary antibody. Signals were detected using a Luminol substrate solution.

### Small Interference RNA Transfection

H1299 and A549 cells (2 × 10^5^) were seeded into 6-well plates and cultured in a humidified incubator at 37°C and 5% CO_2_ for 12 h. Cells were transfected with a siRNA control and three independent siRNA targeting KDM1A by TransIT LT1(Mirus corporation-). Transfected cells were cultured for 48 h before being used for further experiments. The KDM1A siRNA target sequences were as follows: KDM1A siRNA-1: GCTCGACAGTTACAAAGTT; KDM1A siRNA-2: GTTGGATAATCCAAAGATT; KDM1A siRNA-3: GAAGCTACATCTTACCTTA, and all siRNA sequences were purchased from the Ribobio corporation of Guangzhou.

### Cell Metabolism Determination

The extracellular acidification rate (ECAR), was determined by the Seahorse XF96 Extracellular Flux Analyzer (Agilent Technologies, Santa Clara, CA, USA) according to the manufacturer's protocol. Briefly, H1299 and A549 cells were seeded into 6-well plates and cultured in a humidified incubator at 37°C and 5% CO_2_ for 12 h. Cells treated with ORY1001 (80 and 160 μM) or vehicle alone for 24 h, were then seeded into 96-well cell plates. At the same time, the calibration plate was incubated at 37°C, in a non-CO_2_ incubator for 12 h. To determine the cellular aerobic glycolysis profile, ECAR was estimated after sequential injections of glucose (10 mM), oligomycin (1 μM), and 2-DG (100 mM). Protein concentration was quantified using the BCA kit to normalize the data and plotted as the mean ± SD.

### Bioinformatics Analysis

The public Gene Expression Omnibus datasets (GSE19804) and the human protein atlas (https://www.proteinatlas.org) dataset were used for bioinformatics analysis. Kaplan-Meier Plotter (http://kmplot.com/analysis/) was used for overall survival.

### Statistical Analysis

Statistical analyses were performed using the Student's *t*-test. All data were obtained from three independent experiments performed in triplicate and were presented as the mean ± SD. Statistical analyses of the KM curve were performed using the Log-Rank test. We considered a *P* < 0.05, to indicate a statistically significant difference.

## Results

### KDM1A Expression Is Elevated in Lung Cancer and Regulates Cell Proliferation

**Figure 1:**
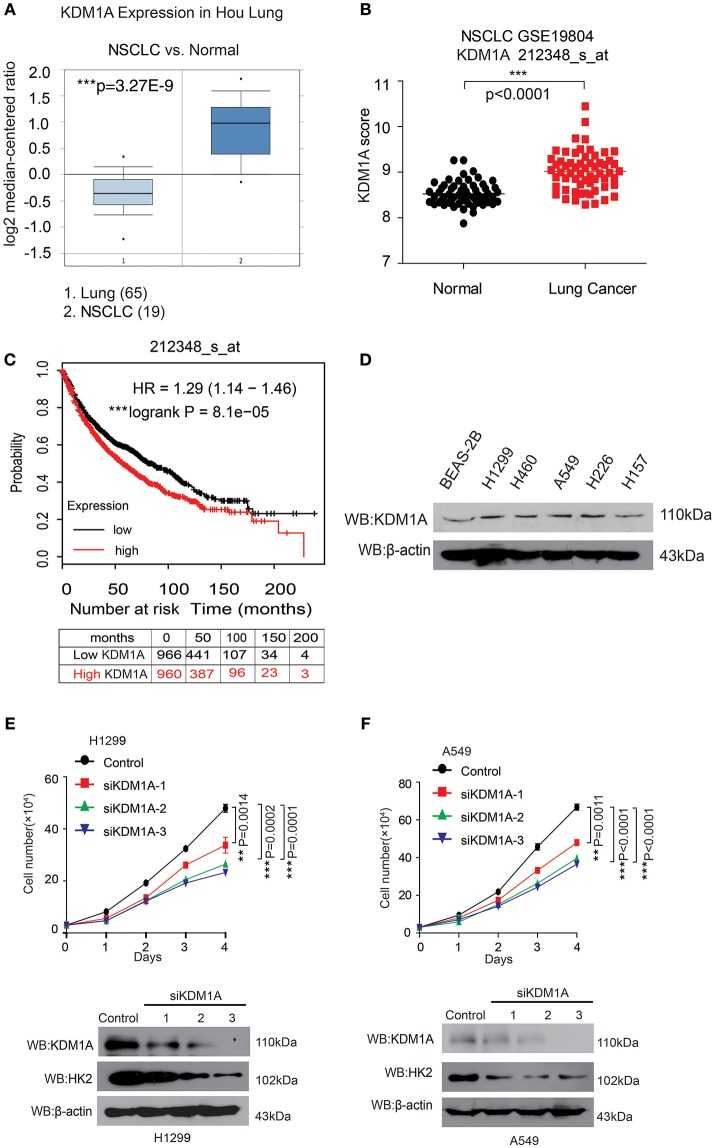
KDM1A expression is evaluated in lung cancer and is important for cancer cell proliferation and tumor growth. **(A)** Oncomine data for the expression of KDM1A in lung and non-small cell lung cancer (NSCLC). **(B)** Expression of KDM1A in lung cancer tissues and matched non-cancerous, calculated from GEO profiles (GSE19804, *n* = 60). **(C)** Kaplan–Meier curves of overall survival in lung cancer patients with high and low expression of KDM1A, calculated from (http://kmplot.com/analysis). **(D)** KDM1A protein levels were analyzed in the majority of a spectrum of diverse human lung cancer cells, including H1944, H460, H1299, H157, H226, A549 cells, and normal proliferating Human Bronchial Epithelial Cell Line (BEAS-2B). **(E,F)** Cell proliferation rates determined by cell counting in human lung cancer H1299 and A549 cells with knockdown of KDM1A. The error bars represent mean values ± SD from three replicates of each sample (^*^: 0.01 < *p* < 0.05; ^**^: 0.001 < *p* < 0.01; ^***^p < 0.001).

In an effort to explore the role of KDM1A in lung cancer, we analyzed the correlation between KDM1A expression levels and the outcomes of lung cancer patients. We first examined the expression of KDM1A in lung cancer tissues using Oncomine data. Figure 1A shows that KDM1A was increased in non-small cell lung cancer (NSCLC) tissue (19) compared with lung tissue (65) (Hou et al., 2010). We also used Gene Expression Omnibus (GEO) profiles (GSE19804) to analyze KDM1A expression and confirmed that the expression of KDM1A was higher in cancer tissues than in non-tumors (Figure 1B). To further substantiate the importance of KDM1A expression in lung cancer progression, we also used the Kaplan-Meier survival analysis in lung cancer patients, based on the publicly available Kaplan-Meier Plotter (http://kmplot.com/analysis/(KDM1A: accession number 212348_s_at). Higher levels of KDM1A (red) are significantly correlated with reduced overall survival compared to low KDM1A levels (black) (Figure 1C). To validate our findings using the publish data, we also checked the expression of KDM1A in the various human lung cancer cells, including H1944, H460, H1299, H157, and H226 cells, compared to the normal proliferating Human Bronchial Epithelial Cell Line (BEAS-2B), but there were not significant differences (Figure 1D).

To determine the role of KDM1A in lung cancer cell proliferation, we found that knockdown of KDM1A by siRNA transient transfection, resulted in decreased cell proliferation in the human lung cancer H1299 and A549 cells (Figures 1E,F). Together these data demonstrated that KDM1A was highly expressed in lung cancer and correlated with overall survival, and that KDM1A plays an important role in cancer cell proliferation, suggesting that KDM1A is a promising anti-cancer target.

### Targeting KDM1A Inhibit Lung Cancer Cell Proliferation and Cell Cycle

**Figure 2:**
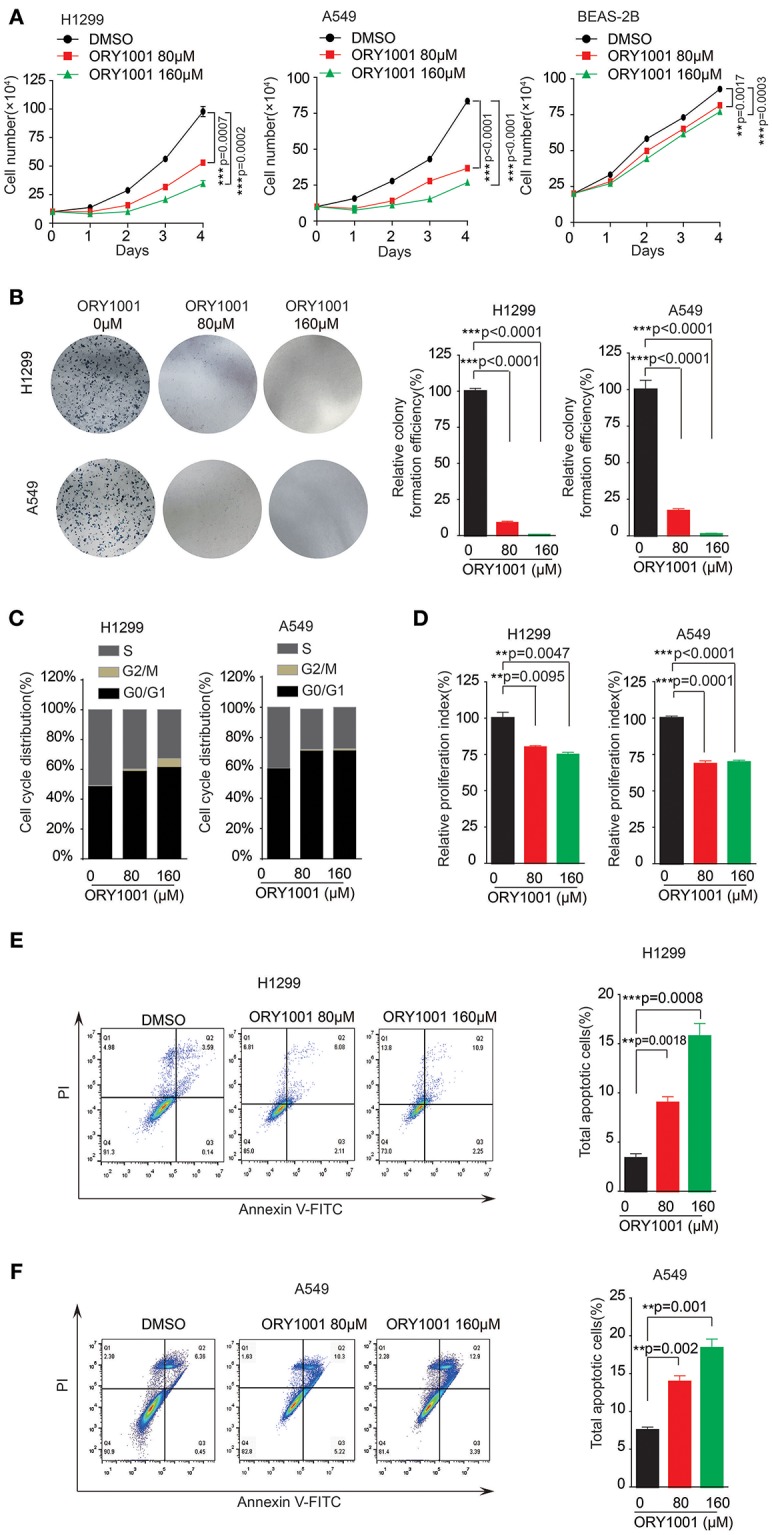
Targeting KDM1A inhibit lung cancer cell proliferation and cell cycle. **(A)** Cell proliferation was determined in H1299, A549, and BEAS-2B cells in the presence of increasing concentrations of ORY-1001. **(B)** Colony formation was determined in H1299 and A549 cells in the presence of increasing concentrations of ORY-1001. **(C,D)** H1299 and A549 cells were treated with increasing concentrations of ORY-1001 for 48 h and stained with propidium iodide (PI) for flow cytometer analysis. **(C)** Bar charts of the cell cycle phases from three independent experiments were shown. **(D)** Cell proliferation index (PI) was calculated based on the indicated equation and is shown. **(E)** H1299 cells were treated with ORY-1001 at the indicating concentrations for 48 h, and then labeled with annexin V-FITC and PI for flow cytometer analysis. A set of representative dot plots of H1299 flow cytometer analysis were shown, and the bar charts show total apoptotic cells from three independent experiments. **(F)** A549 cells were treated with ORY-1001 at the indicating concentrations for 48 h, and then labeled with annexin V-FITC and PI for flow cytometer analysis. A set of representative dot plots of A549 flow cytometer analysis were shown, and the bar charts show total apoptotic cells from three independent experiments. The error bars represent mean values ± SD from three replicates of each sample (^*^: 0.01 < *p* < 0.05; ^**^: 0.001 < *p* < 0.01; ^***^p < 0.001).

ORY-1001 was identified as a potent and selective covalent inhibitor of KDM1A for the inhibition cell proliferation of acute leukemia. In this study, we explored the role of ORY-1001 in lung cancer. Firstly, ORY-1001 treatment resulted in a decreased cell proliferation of lung cancer cells including H1299 and A549 cells, in a time and dose-dependent manner, but did not significantly affect normal BEAS-2B cell proliferation (Figure 2A). Secondly, the colony formation assays revealed that the inhibition of KDM1A by ORY-1001 reduced the colony formation of H1299 and A549 cells (Figure 2B). Thirdly, ORY-1001 treatment resulted in the decreased cell cycle of H1299 and A549 cells (Figures 2C,D). Together these data demonstrated that ORY-1001 treatment suppressed lung cancer cell proliferation, colony formation and the cell cycle. To evaluate the anti-survival effect of KDM1A inhibition by ORY-1001, H1299, and A549 cells were treated with ORY-1001 at different concentrations, and apoptosis of the cells were analyzed by flow cytometry. The results revealed that ORY-1001 induced robust apoptosis in H1299 and A549 cells in a dose-dependent manner (Figures 2E,F). Together these results demonstrated that ORY-1001 inhibited lung cancer cell growth and induced apoptosis.

### KDM1A Inhibition by ORY-1001 Affects the Warburg Effect in Lung Cancer Cells

**Figure 3:**
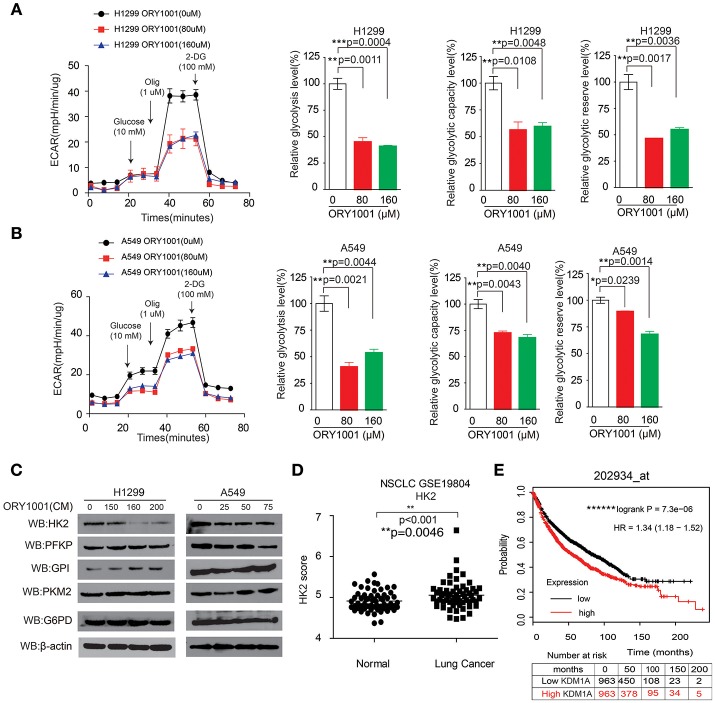
KDM1A inhibition by ORY-1001 affects the Warburg effect in lung cancer cells. **(A,B)** ECAR rate (a proxy for the rate of glycolysis) was used to assess the glycolysis using a Seahorse 96XF extracellular flux analyzer following sequential addition of glucose (10 mM), oligomycin (1.0 μM), and 2-DG (100 mM) as arrows indicated in H1299 and A549 cells in the presence of increasing concentrations of ORY-1001. **(C)** Western blotting assay for the expression of glycolytic enzymes in H1299 and A549 cells in the presence of ORY-1001. **(D)** Expression of HK2 in lung cancer tissues and matched non-cancerous, calculated from GEO profiles (GSE19804, *n* = 60). **(E)** Kaplan–Meier curves of overall survival in lung cancer patients with high and low expression of HK2, calculated from (http://kmplot.com/analysis/). The error bars represent mean values ± SD from three replicates of each sample (^*^: 0.01 < *p* < 0.05; ^**^: 0.001 < *p* < 0.01; ns, not significant).

Next, we attempted to verify whether the effect of KDM1A inhibition by ORY-1001 on the Warburg effect, involved lung cancer cell proliferation and apoptosis. KDM1A inhibition by ORY-1001 decreased glycolysis in H1299 and A549 cells (Figures 3A,B). We further assessed the various parameters of the glycolysis function by analyzing ECAR data at each time point. Our results showed that glycolysis, glycolytic capacity and the glycolytic reserve were markedly decreased in H1299 and A549 cells treated with ORY-1001 (Figures 3A,B). In addition, the protein levels of HK2, were decreased in the ORY-1001 treated cells (Figure 3C). These results suggest that ORY-1001 decreased glycolysis in lung cancer cells by decreasing HK2 expression.

In an effort to determine the role of HK2 in lung cancer mediated by KDM1A, we analyzed the correlation between HK2 expression levels and the outcomes of lung cancer patients. We also analyzed HK2 expression utilizing Gene Expression Omnibus (GEO) profiles (GSE19804) (Figure 3D). To further substantiate the importance of HK2 expression in lung cancer progression, we also used the Kaplan-Meier survival analysis in lung cancer patients based on the publicly available Kaplan-Meier Plotter (http://kmplot.com/analysis/) (HK2: accession number 202934_at). Patients with higher HK2 levels (red) were significantly correlated with reduced overall survival compared to patients with lower HK2 levels (black) (Figure 3E). Together, these data suggest that ORY-1001 affects the Warburg effect in lung cancer cells by targeting KDM1A, leading to the regulation of HK2 expression.

### ORY-1001 Affects Lung Cancer Cell Proliferation and Apoptosis Through Regulating HK2 Expression

**Figure 4:**
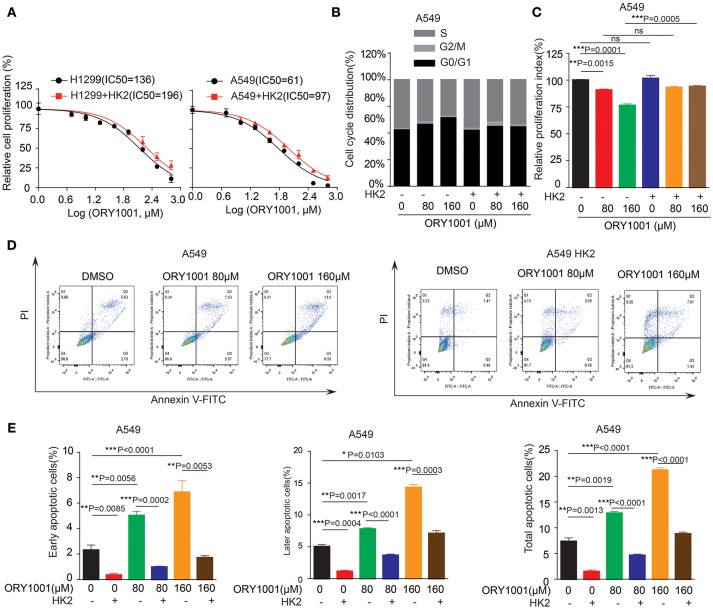
Overexpress HK2 would rescue the effect of ORY-1001 on cells proliferation, cell cycle and apoptosis. **(A)** Cell proliferation was determined in H1299 and A549 cells in the presence of increasing concentrations of ORY-1001 when overexpress HK2. **(B,C)** HK2 overexpress A549 cells were treated with increasing concentrations of ORY-1001 for 48 h and stained with propidium iodide (PI) for flow cytometer analysis. **(B)** Bar charts of the cell cycle phases from three independent experiments were shown. **(C)** Cell proliferation index (PI) was calculated based on the indicated equation and is shown. **(D,E)** HK2 overexpress A549 cells were treated with ORY-1001 at the indicating concentrations for 48 h, and then labeled with annexin V-FITC and PI for flow cytometer analysis. **(D)** A set of representative dot plots of A549 flow cytometer analysis were shown, **(E)** The apoptosis bar charts from three independent experiments were shown. The error bars represent mean values ± SD from three replicates of each sample (^*^: 0.01 < *p* < 0.05; ^**^: 0.001 < *p* < 0.01; ^***^: *p* < 0.001).

We wanted to explore the mechanism of how ORY-1001 inhibits lung cancer cell proliferation and induces apoptosis. Firstly, ORY-1001 treatment resulted in decreased cell proliferation of lung cancer cells, including H1299 and A549 cells, in a time and dose-dependent manner, but this effect was rescued in H1299 and A549 cells, when HK2 was over expressed (Figure 4A). At the same time, the decreased cell cycle of lung cancer cells was also rescued by the overexpression of HK2 (Figures 4B,C). Thirdly, ORY-1001 induced robust apoptosis in H1299 and A549 cells, in a dose-dependent manner and was also rescued by the overexpression of HK2 (Figures 4D,E). Together, these results suggest that ORY-1001 affects lung cancer cell proliferation and apoptosis through regulating HK2 expression.

## Discussion

**Figure 5:**
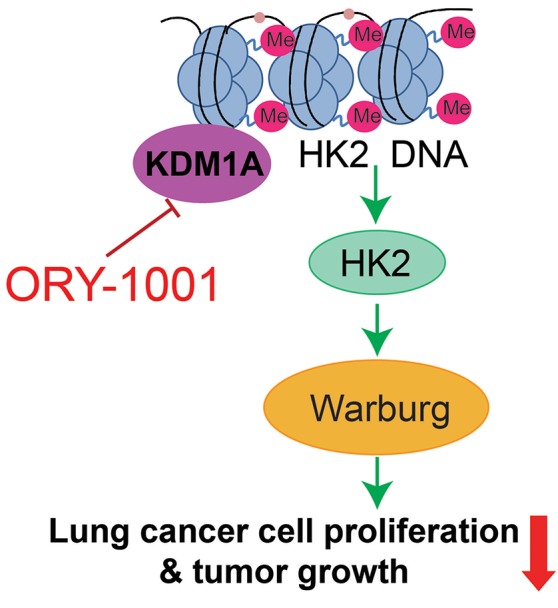
Proposed working model. Schematic model shows that the role and mechanism of ORY-1001 in cancer cell metabolism.

The Warburg effect describes cancer cells with high rates of aerobic glycolysis and increased glucose uptake, which are hallmarks of cancer (Cairns et al., 2011; Zheng et al., 2017; Zhong et al., 2018). The increased aerobic glycolysis rate gives cancer cells a growth advantage, which provides energy and intermediates the rapid growth of a cancer cell. In this study, we established the KDM1A-HK2 axis as a critical regulatory pathway in regulating the Warburg effect. KDM1A directly promotes the expression of key glycolytic genes that facilitate the Warburg effect, which involves the regulation of cell proliferation, cell cycle and apoptosis. Our data indicated that KDM1A is a key factor in the regulation of the Warburg effect and also indicated the causal role of KDM1A in glycolysis regulation mediated by HK2 expression (Figure 5).

Cancer metabolism has been extensively studied, and recent studies have suggested that targeting the enzymes in the metabolic pathways for cancer therapy, seems appealing at first glance. Regulation of cell metabolism to support all fundamental cellular activities is essential for the maintenance of cellular redox homeostasis, reactive oxygen species, growth, proliferation and migration. In the current study, we demonstrated that KDM1A is commonly upregulated in lung cancer and is important for cell proliferation. Using different lung cancer cell lines, we showed that targeting KDM1A by ORY-1001 can facilitate cell proliferation, cell cycle and apoptosis. For the first time we revealed the role that targeting KDM1A plays in controlling cell proliferation, the cell cycle and the role it place in inducing apoptosis, through the promotion of the Warburg effect by regulating the expression of the key enzyme HK2, which is necessary for cell proliferation and rapidly growing tumors. However, we did not exclude the possibility that KDM1A directly demethylates the enzymes in the glycolysis pathway, regulating the Warburg effect by controlling their enzyme activity.

From a translational point of view, therapeutic targeting of KDM1A might be an effective therapeutic option for the advanced growth of lung cancer, as it is sufficient to block the glycolysis signaling pathway. Taken together, our data clearly indicated that targeting the KDM1A would rewire the Warburg effect and inhibit cancer cell proliferation, by regulating the key enzyme HK2 expression.

## Author Contributions

ZL and XZ performed and analyzed all the experiments. JL and LL drafted the work for important intellectual content. YG edited the language and figures. SZ and CS wrote the manuscript and designed the study.

### Conflict of Interest Statement

The authors declare that the research was conducted in the absence of any commercial or financial relationships that could be construed as a potential conflict of interest.

## References

[B1] Cairns R. A. Harris I. S. Mak T. W. (2011). Regulation of cancer cell metabolism. Nat. Rev. Cancer 11, 85–95. 10.1038/nrc2981 21258394

[B2] Fan J. Shan C. Kang H. B. Elf S. Xie J. Tucker M.. (2014). Tyr phosphorylation of PDP1 toggles recruitment between ACAT1 and SIRT3 to regulate the pyruvate dehydrogenase complex. Mol. Cell 53, 534–548. 10.1016/j.molcel.2013.12.026 24486017 PMC3943932

[B3] Fang J. Ying H. Mao T. Fang Y. Lu Y. Wang H.. (2017). Upregulation of CD11b and CD86 through LSD1 inhibition promotes myeloid differentiation and suppresses cell proliferation in human monocytic leukemia cells. Oncotarget 8, 85085–85101. 10.18632/oncotarget.18564 29156705 PMC5689595

[B4] Fiskus W. Sharma S. Shah B. Portier B. P. Devaraj S. G. T. Liu K.. (2017). Highly effective combination of LSD1 (KDM1A) antagonist and pan-histone deacetylase inhibitor against human AML cells. Leukemia 31:1658. 10.1038/leu.2017.77 28322226

[B5] Hayami S. Kelly J. D. Cho H. S. Yoshimatsu M. Unoki M. Tsunoda T.. (2011). Overexpression of LSD1 contributes to human carcinogenesis through chromatin regulation in various cancers. Int. J. Cancer 128, 574–586. 10.1002/ijc.25349 20333681

[B6] Hou J. Aerts J. Den Hamer B. Van Ijcken W. Den Bakker M. Riegman P.. (2010). Gene expression-based classification of non-small cell lung carcinomas and survival prediction. PLoS ONE 5:e10312. 10.1371/journal.pone.0010312 20421987 PMC2858668

[B7] Jie D. Zhongmin Z. Guoqing L. Sheng L. Yi Z. Jing W.. (2013). Positive expression of LSD1 and negative expression of E-cadherin correlate with metastasis and poor prognosis of colon cancer. Dig. Dis. Sci. 58, 1581–1589. 10.1007/s10620-012-2552-2 23314859

[B8] Liu H. Liu Y. Zhang J. T. (2008). A new mechanism of drug resistance in breast cancer cells: fatty acid synthase overexpression-mediated palmitate overproduction. Mol. Cancer Ther. 7, 263–270. 10.1158/1535-7163.MCT-07-0445 18281512

[B9] Lv T. Yuan D. Miao X. Lv Y. Zhan P. Shen X.. (2012). Over-expression of LSD1 promotes proliferation, migration and invasion in non-small cell lung cancer. PLoS ONE 7:e35065. 10.1371/journal.pone.0035065 22493729 PMC3320866

[B10] Maes T. Mascaró C. Tirapu I. Estiarte A. Ciceri F. Lunardi S.. (2018). ORY-1001, a potent and selective covalent KDM1A inhibitor, for the treatment of acute leukemia. Cancer Cell 33, 495–511 e412. 10.1016/j.ccell.2018.02.002 29502954

[B11] Magliulo D. Bernardi R. Messina S. (2018). Lysine-specific demethylase 1A as a promising target in acute myeloid leukemia. Front. Oncol. 8:255. 10.3389/fonc.2018.00255 30073149 PMC6060236

[B12] Sakamoto A. Hino S. Nagaoka K. Anan K. Takase R. Matsumori H.. (2015). Lysine demethylase LSD1 coordinates glycolytic and mitochondrial metabolism in hepatocellular carcinoma cells. Cancer Res. 75, 1445–1456. 10.1158/0008-5472.CAN-14-1560 25649769

[B13] Schildhaus H. U. Riegel R. Hartmann W. Steiner S. Wardelmann E. Merkelbach-Bruse S.. (2011). Lysine-specific demethylase 1 is highly expressed in solitary fibrous tumors, synovial sarcomas, rhabdomyosarcomas, desmoplastic small round cell tumors, and malignant peripheral nerve sheath tumors. Hum. Pathol. 42, 1667–1675. 10.1016/j.humpath.2010.12.025 21531005

[B14] Sehrawat A. Gao L. Wang Y. Bankhead A. III. Mcweeney S. K. King C. J.. (2018). LSD1 activates a lethal prostate cancer gene network independently of its demethylase function. Proc. Natl. Acad. Sci. U. S. A. 115, E4179–E4188. 10.1073/pnas.1719168115 29581250 PMC5939079

[B15] Wang J. B. Erickson J. W. Fuji R. Ramachandran S. Gao P. Dinavahi R.. (2010). Targeting mitochondrial glutaminase activity inhibits oncogenic transformation. Cancer Cell 18, 207–219. 10.1016/j.ccr.2010.08.009 20832749 PMC3078749

[B16] Yu Y. Wang B. Zhang K. Lei Z. Guo Y. Xiao H.. (2013). High expression of lysine-specific demethylase 1 correlates with poor prognosis of patients with esophageal squamous cell carcinoma. Biochem. Biophys. Res. Commun. 437, 192–198. 10.1016/j.bbrc.2013.05.123 23747727

[B17] Zhao Y. Butler E. B. Tan M. (2013). Targeting cellular metabolism to improve cancer therapeutics. Cell Death Dis. 4:e532. 10.1038/cddis.2013.60 23470539 PMC3613838

[B18] Zhao Y. Liu H. Liu Z. Ding Y. Ledoux S. P. Wilson G. L.. (2011). Overcoming trastuzumab resistance in breast cancer by targeting dysregulated glucose metabolism. Cancer Res. 71, 4585–4597. 10.1158/0008-5472.CAN-11-0127 21498634 PMC3129363

[B19] Zhao Z. K. Yu H. F. Wang D. R. Dong P. Chen L. Wu W. G.. (2012). Overexpression of lysine specific demethylase 1 predicts worse prognosis in primary hepatocellular carcinoma patients. World J. Gastroenterol. 18, 6651–6656. 10.3748/wjg.v18.i45.6651 23236241 PMC3516205

[B20] Zheng W. Feng Q. Liu J. Guo Y. Gao L. Li R.. (2017). Inhibition of 6-phosphogluconate dehydrogenase reverses cisplatin resistance in ovarian and lung cancer. Front. Pharmacol. 8:421. 10.3389/fphar.2017.00421 28713273 PMC5491617

[B21] Zhong X. Y. Yuan X. M. Xu Y. Y. Yin M. Yan W. W. Zou S. W.. (2018). CARM1 methylates GAPDH to regulate glucose metabolism and is suppressed in liver cancer. Cell Rep. 24, 3207–3223. 10.1016/j.celrep.2018.08.066 30232003

[B22] Zhou M. Zhao Y. Ding Y. Liu H. Liu Z. Fodstad O.. (2010). Warburg effect in chemosensitivity: targeting lactate dehydrogenase-a re-sensitizes taxol-resistant cancer cells to taxol. Mol. Cancer 9:33. 10.1186/1476-4598-9-33 20144215 PMC2829492

